# Une papule rose de la tempe

**DOI:** 10.11604/pamj.2017.27.83.10917

**Published:** 2017-06-02

**Authors:** Hasnaa Zaouri, Baderdine Hassam

**Affiliations:** 1 Service de Dermatologie-Vénéréologie, Centre Hospitalier Universitaire Avicenne, Université Mohammed V, Rabat, Maroc

**Keywords:** Maladie de Bowen, papule de la tempe, biopsie temporale, Bowen’s disease, papule on the temple, temporal biopsy

## Image en médecine

La maladie de Bowen (MB) est un carcinome épidermoïde in situ relativement rare. Les facteurs favorisant sont l’exposition solaire, les rayonnements ionisants, l’arsenic, l’infection par le virus du papillome humain HPV, certaines génodermatoses, elle peut survenir sur des lésions cutanées chroniques notamment des kératoses actiniques ou de lupus discoïde. Aucun cas de maladie de Bowen au niveau du site de biopsie temporale n’a été rapporté dans la littérature. Nous rapportons le cas d’un patient âgé de 72 ans, de phototype IIIa, suivi depuis une année pour artérite de Horton, sous corticothérapie par voie orale et antiagrégants plaquettaires, qui rapporte depuis six mois, l’apparition d’une papule asymptomatique au niveau du site de la biopsie temporale, augmentant progressivement de taille. L'examen dermatologique trouve une papule de 0,5 cm de taille, de couleur rose-jaune, à surface légèrement kératosique, non infiltrée, siégeant au niveau de la tempe droite, au site de la biopsie temporale. Une biopsie exérèse complète réalisée, a montré un revêtement épidermique, acanthosique et parakératosique présentant une désorganisation architecturale sur toute la hauteur avec présence d’atypies cytonucléaires marquées et mitoses, le derme sous jacent comporte un infiltrat inflammatoire modéré péri vasculaire, en faveur de la MB. Le patient a bénéficié d’une séance de cryothérapie à l’azote liquide et il a était mis sous crème à base de 5 fluoro-uracile pendant un mois avec photo protection. Le pronostic de la MB est en général favorable. En revanche, en l'absence d'un diagnostic et d'un traitement approprié, l’évolution se fait vers un véritable carcinome épidermoïde invasif.

**Figure 1:**
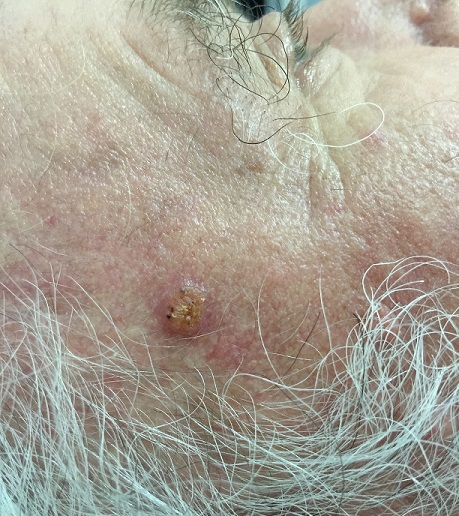
Papule rose-jaune, légèrement kératosique de la tempe droite

